# Effect of vosoritide on genu varum in children with achondroplasia after 1 year in randomized placebo-controlled trials

**DOI:** 10.1210/jendso/bvag024

**Published:** 2026-02-06

**Authors:** Klane K White, Melita Irving, Swati Mukherjee, Christine Rivat, Ian Sabir, Anne Dee, Ravi Savarirayan

**Affiliations:** Children's Hospital Colorado, Aurora, CO 80045, USA; Guy's and St. Thomas' NHS Foundation Trust, Evelina Children's Hospital, London SE1 7EH, UK; BioMarin (UK) Ltd, London WC1A 2SL, UK; BioMarin (UK) Ltd, London WC1A 2SL, UK; BioMarin (UK) Ltd, London WC1A 2SL, UK; BioMarin Pharmaceutical Inc., Novato, CA 94949, USA; Murdoch Children's Research Institute, Royal Children's Hospital and University of Melbourne, Parkville, Victoria 3052, Australia

**Keywords:** achondroplasia, genu varum, leg bowing, fibular overgrowth, bone, tibia

## Abstract

Achondroplasia is a skeletal dysplasia associated with multisystem complications including genu varum, which causes pain and limits mobility. Vosoritide, a targeted treatment for achondroplasia, improves growth in children and has an established safety profile, but its effect on genu varum is unclear. Data were collected from 183 participants from randomized, double-blind, placebo-controlled phase 2 (CANOPY ACH-2I [111-206; NCT03583697]) and phase 3 (CANOPY ACH-3 [111-301; NCT03197766]) studies evaluating vosoritide (weight-based dose of 15 or 30 µg/kg/day) in children aged 0-5 and >5 years, respectively. Anterior/posterior lower limb radiographs were taken at baseline and 1 year to measure parameters associated with genu varum. Differences in least-squares mean (LSM) change from baseline were calculated for vosoritide vs placebo using an analysis of covariance model. In both studies, age at treatment initiation and sex distribution were generally balanced between vosoritide and placebo (except 58% males received vosoritide in CANOPY ACH-2I). After 1 year of vosoritide treatment vs placebo, tibial bowing decreased in children who initiated treatment aged <5 years (*n* = 40) and remained stable in those ≥5 years (*n* = 57). Vosoritide-treated participants with evidence of abnormal fibular growth at baseline had reduced overall and distal fibular overgrowth vs placebo. Children aged ≥5 years improved in ankle joint to distal fibula physis distance (difference in LSM change from baseline, −0.07, *P* = .010) and fibula/tibia ratio (−0.025, *P* < .0001), and children aged 2-5 years had improved fibula/tibia ratio (−0.033, *P* = .0032). Preliminary results suggest vosoritide may improve or limit genu varum in children with achondroplasia.

Achondroplasia is an inherited skeletal dysplasia condition caused by gain-of-function variants in fibroblast growth factor receptor 3 (*FGFR3*) and resulting in overactive FGFR3 signaling leading to impaired endochondral bone growth. The condition is characterized by medical and functional complications that impact health-related quality of life (HRQOL) of individuals with achondroplasia [1-3]. In addition to disproportionate short stature, other clinical manifestations of achondroplasia include rhizomelia, spinal deformities, and skeletal deformities of the extremities, including genu varum [3].

Genu varum occurs in 40% to 70% of children with achondroplasia and often persists into adulthood [2, 4]. It is frequently a source of leg pain and impaired function and potentially impacts gait and mobility [2, 5]. Approximately 25% of children with achondroplasia require corrective realignment surgery (ie, hemiepiphysiodesis and/or osteotomy) [5, 6]. Though often referred to as genu varum or leg bowing, this achondroplasia-associated skeletal deformity of the lower extremities is complex and involves the distal femur, fibular overgrowth, lateral dynamic instability of the knee, proximal and distal tibial varus, internal tibial torsion, and tibial recurvatum [5, 7].

Tibial bowing is anatomically characterized by a nonparallel relationship between the distal and proximal physes in the lower leg [8] and the resulting angle referred to as the tibial bowing angle [8]. Genu varum is also believed to be associated with overgrowth of the fibula [5]. In one foundational study, the fibula length of children with achondroplasia between 0 and 15 years of age was found to be consistently greater than the tibia length, resulting in a fibula/tibia (*F*/*T*) ratio of 1.08, compared with 0.98 in an average-stature population [8]. Distal fibular overgrowth may result in an increased distance between the distal fibular physis and the ankle mortise, affecting the stability of the ankle joint and potentially contributing to bowing of the legs [8].

Vosoritide is a C-type natriuretic peptide analog approved for the treatment of children with achondroplasia from infancy until the closure of the epiphyses [9, 10]. Vosoritide downregulates overactive FGFR3 signaling to stimulate endochondral bone growth. An extensive clinical program and growing real-world evidence demonstrate that vosoritide improves growth, including height gain; positively impacts body proportionality and HRQOL; and reduces common complications associated with achondroplasia [11-13]. While previous studies have assessed the effect of vosoritide on overall growth and functionality [14-16], clinical evidence of the impact of vosoritide treatment on genu varum has so far been limited.

In this analysis, we compare key bone morphology parameters of the tibia and fibula in participants treated with vosoritide or placebo at baseline and after 1 year in the CANOPY ACH-2I (111-206) and CANOPY ACH-3 (111-301) studies to evaluate the potential impact of vosoritide on genu varum in children with achondroplasia.

## Materials and methods

### Study design

**Figure 1:**
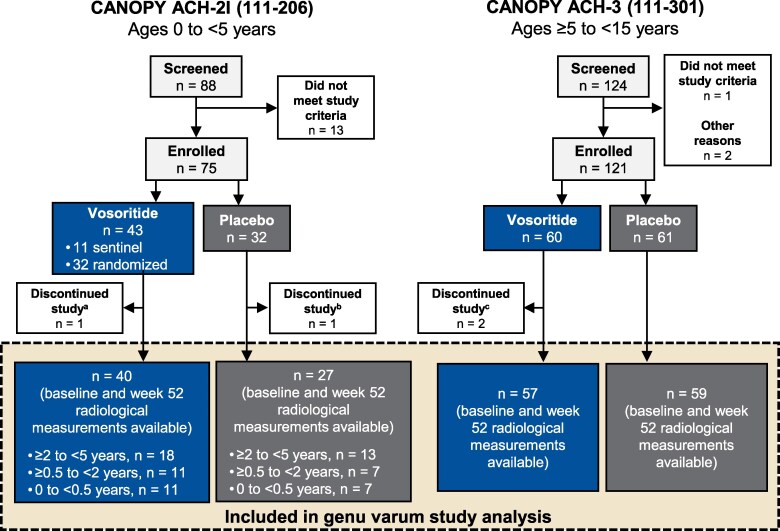
Combined CONSORT diagram depicting study participants from phase 2 CANOPY ACH-2I (111-206) and phase 3 CANOPY ACH-3 (111-301) clinical trials. ^a^Participant discontinued from study due to a fatal AE assessed as not related to study treatment. ^b^Study discontinuation was due to withdrawal by participant. ^c^One participant discontinued from study due to an AE (anxiety about injections), and the other discontinued due to withdrawal by participant. Abbreviation: AE, adverse event.

Participants for this secondary analysis were from the 1-year, randomized, double-blind, placebo-controlled phase 2 CANOPY ACH-2I (111-206; NCT03583697, *N* = 75) [15] and phase 3 CANOPY ACH-3 (111-301; NCT03197766, *N* = 121) [16] studies that evaluated the safety and efficacy of vosoritide in children with achondroplasia (Fig. 1). Radiograph data at baseline and 1-year follow-up were available for 67 participants from CANOPY ACH-2I and 116 participants from CANOPY ACH-3. Participants received daily subcutaneous injections of vosoritide, 15 and/or 30 µg/kg in CANOPY ACH-2I and 15 µg/kg in CANOPY ACH-3. Detailed eligibility criteria have been published previously and include genetic confirmation of achondroplasia. Exclusion criteria common to both studies included hypochondroplasia or other short-stature conditions, any evidence of cervicomedullary compression likely to require surgical intervention, and any planned limb-lengthening procedures during the study period [15, 16]. Participants with prior limb-lengthening surgery were excluded from CANOPY ACH-2I, but participants were still able to enroll in CANOPY ACH-3 if the previous surgery occurred at least 18 months prior to screening and the participant healed without sequelae. No participants had leg alignment surgery during the 1-year study period. At treatment initiation, participants from CANOPY ACH-2I were <5 years old and those from CANOPY ACH-3 were ≥5 to <15 years old.

### Outcome assessments

Anterior/posterior lower limb radiographs were taken for all participants enrolled in both CANOPY studies at baseline and at the 1-year follow-up. Image acquisition was standardized across the study sites, and radiographs were centrally read by independent radiologists to minimize variability in measurement.

**Figure 2:**
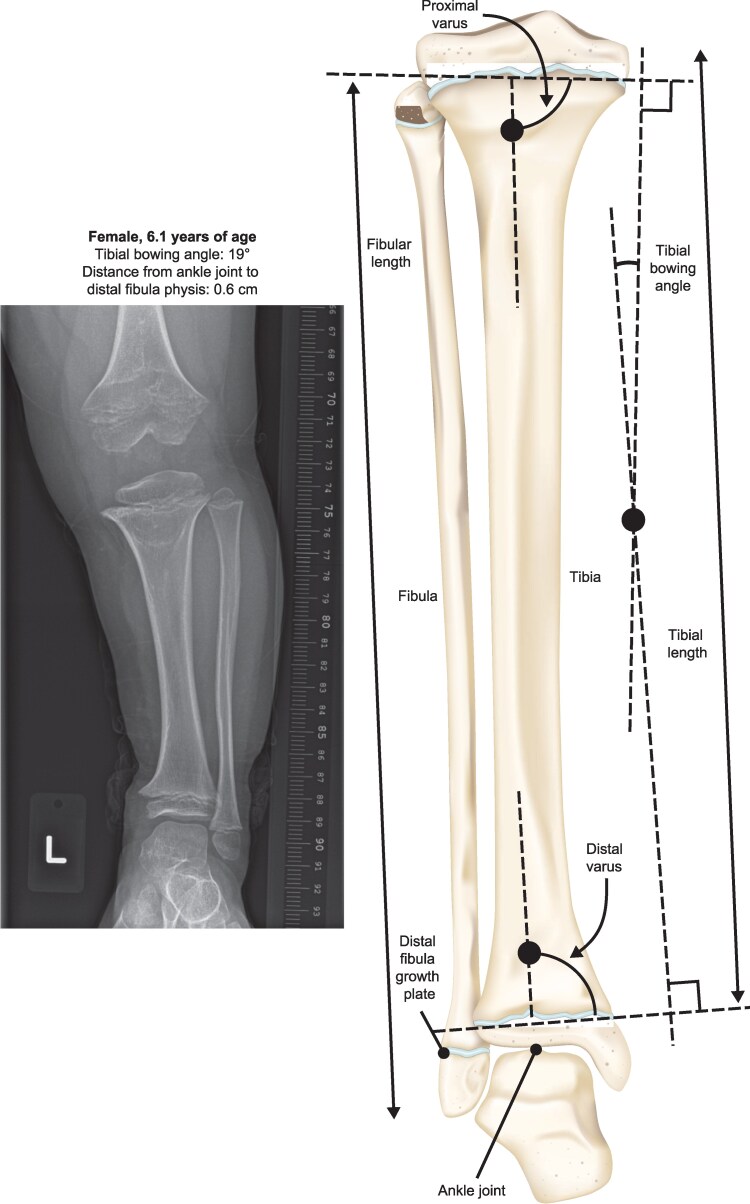
Representative lower left leg radiograph displaying untreated genu varum in a child with achondroplasia and a schematic of lower leg bone morphology parameters in a child of average stature.

Morphology of lower leg bones was assessed using radiographic measurements of 3 parameters: tibial bowing angle (degrees), *F*/*T* ratio (fibula length [cm]/tibia length [cm]), and distance from the top of the ankle mortise/joint to distal fibula physis (cm), where increased distance is indicative of distal fibular overgrowth (Fig. 2). The degree of tibial bowing was observed radiographically as the linear intersections derived from the perpendicular of the upper and lower physes. Measurements for these 3 bone morphology parameters were collected at baseline and after 1 year of vosoritide or placebo treatment for both legs.

### Ethics statement

These studies were conducted according to the Declaration of Helsinki, and the independent ethics committee or institutional review boards of all study sites approved the study protocols. The legally authorized representatives (parents/guardians) of all participants provided written informed consent.

### Statistics

Data are reported as means (SD). The overall (combined left and right leg) measurements for participants were reported by study (CANOPY ACH-2I: 0 to <5 years; CANOPY ACH-3: ≥5 to <15 years) and by age intervals (0 to <2 years, ≥2 to <5 years, and ≥5 to <15 years) to account for baseline differences between age groups.

The magnitude of change in the bone morphology parameters was quantified as least-squares mean (LSM) change from baseline derived from a repeated-measures analysis of covariance model with 95% confidence intervals (CIs) for treatment difference and corresponding 2-sided *P*-values. Model terms included treatment, sex, Tanner stage (CANOPY ACH-3 only), age stratum (CANOPY ACH-2I only), baseline age, baseline annualized growth velocity (AGV), baseline height Z-score, and baseline bone morphology parameter. Adjustment was made for both age stratum and baseline age, as the CANOPY ACH-2I study design had a staggered, age-descending recruitment of 3 age cohorts based on age at study screening. The difference in LSM change from baseline was calculated as vosoritide minus placebo.

## Results

### Participants

Baseline characteristics were comparable between participants who received vosoritide or placebo in CANOPY ACH-2I (placebo, *n* = 27; vosoritide, *n* = 40) and CANOPY ACH-3 (placebo, *n* = 59; vosoritide, *n* = 57), apart from a small imbalance in the proportions of male vs female participants in CANOPY ACH-2I (Table 1). Baseline mean AGV was higher for participants who received vosoritide vs placebo in both studies, and height Z-score was lower for participants who received vosoritide vs placebo in CANOPY ACH-2I. Participants from both studies were predominantly White and not Hispanic or Latino. Mean ages at treatment initiation were similar between participants assigned to vosoritide and placebo from both studies. At baseline, most CANOPY ACH-3 participants were at Tanner stage I.

**Table 1:** Participant demographics and baseline characteristics

Characteristic	Treatment	
	Placebo	Vosoritide
**CANOPY ACH-2I (111-206): 0 to <5 years, *N***	27	40
Age at treatment initiation (years)		
Mean (SD)	2.15 (1.54)	2.05 (1.48)
Sex, *n* (%)		
Male	12 (44.4)	23 (57.5)
Female	15 (55.6)	17 (42.5)
Race, *n* (%)		
White	21 (77.8)	27 (67.5)
Asian	6 (22.2)	11 (27.5)
Multiple	0	2 (5.0)
Ethnicity, *n* (%)		
Not Hispanic or Latino	24 (88.9)	37 (92.5)
Hispanic or Latino	3 (11.1)	3 (7.5)
AGV (cm/year)		
Mean (SD)	9.84 (8.18)	11.69 (7.72)
Height Z-score*^a^*		
Mean (SD)	−4.29 (1.52)	−3.87 (0.91)
**CANOPY ACH-3 (111-301): ≥5 to <15 years, *N***	59	57
Age at treatment initiation (years)		
Mean (SD)	8.98 (2.39)	8.29 (2.43)
Sex, *n* (%)		
Male	33 (55.9)	29 (50.9)
Female	26 (44.1)	28 (49.1)
Race, *n* (%)		
White	39 (66.1)	44 (77.2)
Asian	13 (22.0)	9 (15.8)
Multiple	5 (8.5)	2 (3.5)
Black or African American	2 (3.4)	2 (3.5)
Ethnicity, *n* (%)		
Not Hispanic or Latino	53 (89.8)	56 (98.2)
Hispanic or Latino	6 (10.2)	1 (1.8)
Tanner stage, *n* (%)*^b^*		
I	47 (79.7)	46 (80.7)
>I	12 (20.3)	11 (19.3)
AGV (cm/year)		
Mean (SD)	4.06 (1.20)	4.28 (1.54)
Height Z-score*^a^*		
Mean (SD)	−5.11 (1.02)	−5.10 (1.12)

Abbreviation: AGV, annualized growth velocity.

^*a*^Z-scores were derived using age- and sex-specific reference data (means and SDs) for average-stature children per the Centers for Disease Control and Prevention.

^*b*^Tanner stage (I, >I) is determined using the genitalia and breast Tanner stage for male and female participants, respectively.

Analysis only includes participants who provided both baseline and 52-week genu varum parameter measurements.

### Tibial bowing angle

**Figure 3:**
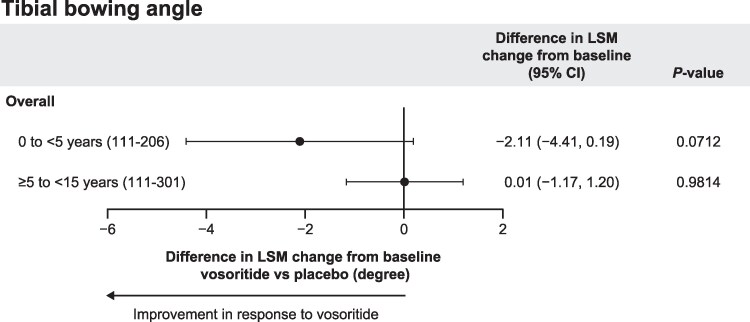
Difference in LSM change from baseline of tibial bowing angle in children treated for 1 year with vosoritide vs placebo with ANCOVA-adjusted differences. Children aged 0 to <5 years were from CANOPY ACH-2I (111-206), and children ages ≥5 to <15 years were from CANOPY ACH-3 (111-301). Overall denotes combined left and right leg data. Abbreviations: ANCOVA, analysis of covariance; CI, confidence interval; LSM, least-squares mean.

At baseline, the mean (SD) tibial bowing angles for participants receiving placebo and vosoritide were 11.3° (6.7°) and 11.1° (7.0°) in CANOPY ACH-2I (participants aged 0 to <5 years) and 8.2° (5.8°) and 8.3° (5.3°) in CANOPY ACH-3 (participants aged ≥5 to <15 years), respectively (Table 2). After 1 year, the mean tibial bowing angle decreased in the vosoritide-treated participants aged <5 years at treatment initiation (LSM change from baseline [95% CI]: −1.8° [−3.2°, −0.4°]), while those who received placebo had a small increase (LSM change from baseline [95% CI]: 0.3° [−1.4°, 2.0°]). Overall, the difference in LSM change from baseline (95% CI) was −2.11 (−4.4, 0.2) in response to vosoritide after 1 year of treatment in children <5 years of age at treatment initiation (*P* = .071; Fig. 3). There was no difference in tibial bowing angle in vosoritide-treated children aged ≥5 to <15 years at treatment initiation compared with placebo (difference in LSM change from baseline [95% CI]: 0.01 [−1.2, 1.2]; *P* = .98). Tibial bowing angle measurements were comparable between the left and right legs, with trends toward improvement in the younger children treated with vosoritide vs placebo (Table 2).

**Table 2:** Tibial bowing angle (degrees) in children treated for 1 year with vosoritide vs placebo

Age cohort	Placebo				Vosoritide				Difference in LSM change from baseline (95% CI)*^a^*
	*n*	Baseline	Year 1	LSM change from baseline (95% CI)	*n*	Baseline	Year 1	LSM change from baseline (95% CI)	
Overall									
0 to <5 years	27	11.3 (6.7)	11.4 (8.0)	0.3 (−1.4, 2.0)	39	11.1 (7.0)	9.4 (6.3)	−1.8 (−3.2, −0.4)	−2.1 (−4.4, 0.2)
≥5 to <15 years	59	8.2 (5.8)	8.7 (6.7)	0.5 (−0.3, 1.3)	57	8.3 (5.3)	8.8 (5.8)	0.5 (−0.3, 1.3)	0.01 (−1.2, 1.2)
Left leg									
0 to <5 years	27	12.0 (7.4)	13.1 (8.8)	1.6 (−0.6, 3.8)	39	10.1 (7.3)	9.2 (6.4)	−1.4 (−3.1, 0.4)	−3.0 (−5.9, -0.1)
≥5 to <15 years	59	7.8 (5.2)	8.5 (6.5)	0.6 (−0.5, 1.7)	57	7.6 (5.4)	8.4 (6.2)	0.9 (−0.2, 2.0)	0.3 (−1.3, 1.9)
Right leg									
0 to <5 years	27	10.6 (6.0)	9.7 (6.9)	−1.1 (−3.1, 1.0)	38	12.0 (6.7)	9.7 (6.2)	−2.3 (−4.0, −0.5)	−1.2 (−4.0, 1.6)
≥5 to <15 years	58	8.6 (6.4)	8.9 (7.0)	0.3 (−0.7, 1.3)	57	9.0 (5.2)	9.1 (5.5)	0.1 (−0.9, 1.1)	−0.2 (−1.6, 1.2)

Abbreviations: CI, confidence interval; LSM, least-squares mean.

Mean (SD) or LSM change from baseline (95% CI) are depicted. Overall denotes combined left and right leg data.

^*a*^Difference is vosoritide minus placebo.

### Fibula/tibia ratio

**Figure 4:**
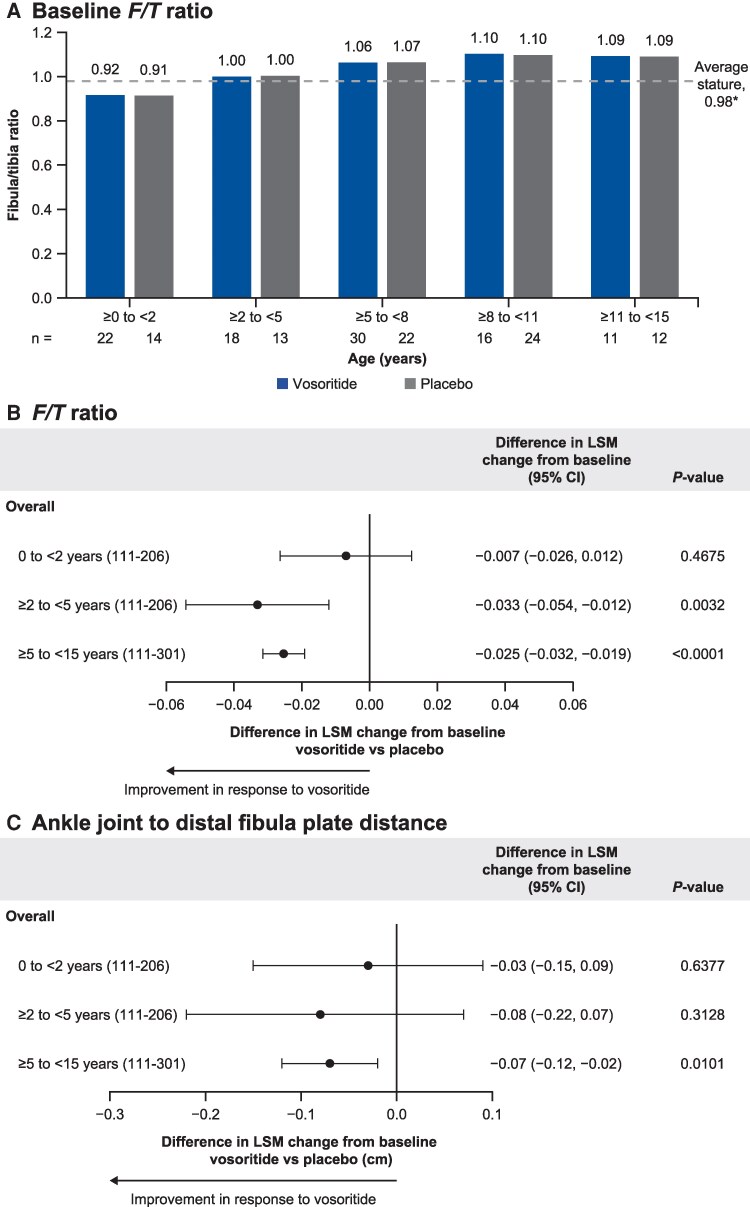
Measured parameters of fibular overgrowth in participants. (A) Baseline *F/T* ratio by age cohorts in untreated children with achondroplasia. Difference in LSM change from baseline in children treated for 1 year with vosoritide vs placebo with ANCOVA-adjusted differences for (B) *F*/*T* ratio and (C) ankle joint to distal fibula plate distance. *F/*T* ratio in children of average stature [8]. Children aged 0 to <5 years were from CANOPY ACH-2I (111-206), and children ages ≥5 to <15 years were from CANOPY ACH-3 (111-301). Overall denotes combined left and right leg data. Abbreviations: ANCOVA, analysis of covariance; CI, confidence interval; *F*/*T*, fibula/tibia; LSM, least-squares mean.

The *F*/*T* ratio was measured at baseline and 1 year for all participants from both CANOPY studies. Before treatment, participants assigned to vosoritide and placebo had similar baseline *F*/*T* ratios, with the ratio generally increasing with age (Fig. 4A). Contrary to previous reports, the tibia was found to be generally longer than the fibula at baseline in participants aged <2 years, with a mean (SD) *F*/*T* ratio of 0.92 (0.05) for participants assigned to vosoritide and 0.91 (0.04) for those assigned to placebo (Table 3). A ratio above that established from an average-stature population (0.98) was only observed in untreated participants aged ≥2 years.

**Table 3:** *F*/*T* ratio in children treated for 1 year with vosoritide vs placebo

Age cohort	Placebo				Vosoritide				Difference in LSM change from baseline (95% CI)*^a^*
	*n*	Baseline	Year 1	LSM change from baseline (95% CI)	*n*	Baseline	Year 1	LSM change from baseline (95% CI)	
Overall									
0 to <2 years	14	0.91 (0.04)	0.95 (0.03)	0.03 (0.01, 0.04)	22	0.92 (0.05)	0.94 (0.05)	0.02 (0.01, 0.03)	−0.007 (−0.03, 0.01)
≥2 to <5 years	13	1.00 (0.04)	1.03 (0.04)	0.04 (0.02, 0.05)	18	1.00 (0.04)	1.01 (0.05)	0.00 (−0.01, 0.02)	−0.03 (−0.05, −0.01)
≥5 to <15 years	58	1.08 (0.04)	1.10 (0.04)	0.01 (0.01, 0.02)	57	1.08 (0.04)	1.07 (0.04)	−0.01 (−0.02, −0.01)	−0.03 (−0.03, −0.02)
Left leg									
0 to <2 years	14	0.91 (0.04)	0.95 (0.02)	0.03 (0.02, 0.05)	22	0.91 (0.05)	0.94 (0.05)	0.02 (0.01, 0.04)	−0.009 (−0.03, 0.009)
≥2 to <5 years	13	1.00 (0.05)	1.03 (0.05)	0.04 (0.02, 0.06)	18	1.00 (0.04)	1.01 (0.05)	0.01 (−0.01, 0.02)	−0.03 (−0.05, −0.01)
≥5 to <15 years	58	1.08 (0.04)	1.10 (0.04)	0.01 (0.01, 0.02)	57	1.08 (0.04)	1.07 (0.04)	−0.01 (−0.02, −0.01)	−0.03 (−0.03, −0.02)
Right leg									
0 to <2 years	14	0.92 (0.04)	0.94 (0.03)	0.02 (0.00, 0.05)	22	0.92 (0.05)	0.94 (0.05)	0.02 (0.00, 0.04)	−0.005 (−0.03, 0.02)
≥2 to <5 years	13	1.01 (0.04)	1.04 (0.04)	0.03 (0.01, 0.05)	18	1.0 (0.04)	1.00 (0.05)	0.00 (−0.01, 0.02)	−0.03 (−0.05, −0.006)
≥5 to <15 years	58	1.09 (0.04)	1.10 (0.03)	0.01 (0.01, 0.02)	57	1.08 (0.05)	1.07 (0.04)	−0.01 (−0.02, −0.01)	−0.03 (−0.03, −0.02)

Abbreviations: CI, confidence interval; *F*/*T*, fibula/tibia; LSM, least-squares mean.

Mean (SD) or LSM change from baseline (95% CI) are depicted. Overall denotes combined left and right leg data.

^*a*^Difference is vosoritide minus placebo.

A consistent increase in *F*/*T* ratio was observed at year 1 vs baseline in all participants who received placebo (Table 3). Among the vosoritide-treated participants, the ratio still increased in those aged <2 years but generally stabilized around 1.0 in those aged ≥2 to <5 years, while decreasing in older participants aged ≥5 years at treatment initiation. The difference in LSM change from baseline (95% CI) for *F*/*T* ratio showed improvement in response to vosoritide vs placebo in children aged ≥2 years at the start of treatment (≥2 to <5 years: −0.033 [−0.05, −0.01], *P* = .0032; ≥5 to <15 years: −0.0025 [−0.03, −0.02], *P* < .0001; Fig. 4B). Similar trends for improvement were seen in the left and right leg (Table 3).

### Distance from ankle joint to distal fibula physis

The mean distance between the ankle joint and distal fibula physis increased with age at baseline in untreated participants (Table 4). Small differences were observed at baseline between participants aged 0 to <5 years receiving placebo and vosoritide, which were adjusted for in the covariance model. After 1 year, the mean distance increased in participants aged <2 years who received either vosoritide or placebo, compared with baseline. In children aged ≥2 to <5 years at treatment initiation, the distance did not vary over time (baseline to year 1) among participants on placebo (remaining at 0.42 cm) but did decrease with vosoritide treatment (from 0.53 to 0.43 cm, respectively). The difference in LSM change from baseline (95% CI) showed an improvement for children aged ≥2 to <5 years receiving vosoritide vs placebo (−0.08 [−0.22, 0.07], *P* = .31), reaching statistical significance in participants aged ≥5 to <15 years (−0.07 [−0.12, −0.02], *P* = .010; Fig. 4C). Similar trends for improvement were seen in the left and right leg (Table 4).

**Table 4:** Ankle joint to distal fibula physis distance (cm) in children treated for 1 year with vosoritide vs placebo

Age cohort	Placebo				Vosoritide				Difference in LSM change from baseline (95% CI)*^a^*
	*n*	Baseline	Year 1	LSM change from baseline (95% CI)	*n*	Baseline	Year 1	LSM change from baseline (95% CI)	
Overall									
0 to <2 years	13	0.15 (0.13)	0.27 (0.20)	0.07 (−0.02, 0.17)	20	0.27 (0.18)	0.29 (0.18)	0.05 (−0.03, 0.12)	−0.03 (−0.15, 0.09)
≥2 to <5 years	13	0.42 (0.21)	0.42 (0.24)	−0.02 (−0.12, 0.09)	18	0.53 (0.24)	0.43 (0.22)	−0.09 (−0.18, 0.00)	−0.08 (−0.22, 0.07)
≥5 to <15 years	58	0.58 (0.20)	0.60 (0.21)	0.01 (−0.03, 0.05)	57	0.59 (0.24)	0.53 (0.23)	−0.06 (−0.10, −0.02)	−0.07 (−0.12, −0.02)
Left leg									
0 to <2 years	13	0.13 (0.12)	0.27 (0.19)	0.09 (−0.04, 0.23)	20	0.26 (0.15)	0.31 (0.21)	0.08 (−0.03, 0.19)	−0.01 (−0.2, 0.17)
≥2 to <5 years	13	0.42 (0.21)	0.42 (0.26)	−0.01 (−0.12, 0.11)	18	0.52 (0.26)	0.42 (0.22)	−0.11 (−0.20, −0.01)	−0.1 (−0.26, 0.06)
≥5 to <15 years	58	0.59 (0.19)	0.59 (0.21)	−0.01 (−0.05, 0.04)	57	0.61 (0.26)	0.54 (0.25)	−0.06 (−0.10, −0.01)	−0.05 (−0.11, 0.01)
Right leg									
0 to <2 years	13	0.17 (0.14)	0.27 (0.21)	0.05 (−0.04, 0.14)	20	0.28 (0.21)	0.26 (0.16)	0.02 (−0.06, 0.09)	−0.04 (−0.16, 0.09)
≥2 to <5 years	13	0.41 (0.21)	0.42 (0.22)	−0.02 (−0.15, 0.11)	17	0.55 (0.22)	0.45 (0.22)	−0.08 (−0.19, 0.03)	−0.06 (−0.24, 0.12)
≥5 to <15 years	58	0.57 (0.21)	0.61 (0.22)	0.03 (−0.02, 0.07)	57	0.57 (0.23)	0.51 (0.22)	−0.06 (−0.11, −0.02)	−0.09 (−0.15, −0.03)

Abbreviations: CI, confidence interval; LSM, least-squares mean.

Mean (SD) or LSM change from baseline (95% CI) are depicted. Overall denotes combined left and right leg data.

^*a*^Difference is vosoritide minus placebo.

## Discussion

Genu varum is a multifactorial complication of achondroplasia that is caused by skeletal deformities of the leg often associated with ligamentous instability of the knee, resulting in pain, limited functionality, and need for realignment surgery in individuals or children with achondroplasia [3, 5]. Varus deformity of the legs at the level of the knees (distal femur or proximal tibia) or ankle (distal tibia), internal torsion of the tibia/fibula, and overgrowth of the fibula in relation to the tibia are the most common deformities associated with genu varum, but their contribution differs depending on the age of the child. While genu varum is generally associated with overgrowth of the proximal fibula resulting in proximal tibial varus and lateral collateral ligament laxity in younger children ≤6 years of age, overgrowth of the distal fibula and a gradual increase in distal tibia varus have been identified as causal factors in children aged ≥8 years [4, 8].

This secondary analysis of the clinical data from the CANOPY ACH-2I and ACH-3 studies showed a positive impact of vosoritide on several parameters of tibial and fibular morphology, suggesting a potential improvement of genu varum in participants receiving vosoritide continuously for at least 1 year. While not statistically significant, the tibial bowing angle decreased in children aged 0 to <5 years, and no worsening was seen in children aged ≥5 years at treatment initiation. Statistically significant improvements in the *F*/*T* ratio were seen in participants aged ≥2 years, whose baseline ratio was higher than the reported average-stature norm. The distance from the ankle joint to distal fibula physis, a marker of distal fibular growth in older children, also decreased in participants with evidence of fibular overgrowth, reaching statistical significance in children aged ≥5 years. This was consistent with reported evidence of the involvement of the distal fibula in this age group. Improvements in the *F*/*T* ratio suggest a faster growth of the tibia relative to the fibula, reflecting potential catch-up growth of the tibia in affected children. In this study, all data were collected and analyzed separately for the left and right legs, as genu varum can be asymmetric [2]; however, comparable trends were seen on both sides for all evaluated parameters.

The results reported here are supported by recent real-world evidence, including data from a 1-year, open-label prospective study in Japanese children aged 3 to 12 years with achondroplasia [14]. Children who received vosoritide demonstrated improvement in genu varum, including statistically significant decreases in tibiofemoral angle and proximal and distal femur varus, as well as trends toward a decrease in proximal and distal tibia varus [14]. However, as that study assessed different parameters—including the mechanical medial proximal tibial angle and mechanical lateral distal tibial angles—the findings, while supportive in principle of results from this study, cannot be directly compared. This highlights the need for additional research in this area and the importance of standardized methodologies, as several different methods of assessing genu varum exist, including tibial and fibular radiographic evaluations as conducted here, mechanical axis deviation of the knee [17], standardized tibial and femoral angle measurements [18], and gait analysis [3, 14, 19, 20].

This study had several limitations. First, the impact of vosoritide treatment was only assessed across the 1-year placebo-controlled period, precluding the establishment of associations with long-term clinical outcomes such as pain and the incidence of corrective realignment surgery. Additionally, the lack of a direct, quantitatively established degree of leg bowing correlated with clinically significant outcomes makes it difficult to assess the impact of treatment. Long-term data from the ongoing extension studies CANOPY ACH-EXT (111-208 [NCT03989947] and 111-302 [NCT03424018]) [21, 22] may be beneficial but will not allow comparison with the natural progression of lower limb alignment in achondroplasia. Second, although genu varum is known to be multifactorial and involve several bones in the lower extremities, this analysis predominantly focused on the lower leg [5]. The analysis of additional parameters, such as the mechanical axis deviation of the knee and the tibiofemoral angle, may provide supportive information [23]. Third, the study was not designed to evaluate the effect of vosoritide on preventing or reversing lower extremity deformity. No formal hypothesis testing was conducted, and the studies were not powered to detect the impacts of vosoritide on parameters of the tibia or fibula morphology.

In this secondary analysis, tibial bowing angle improved in children who initiated vosoritide treatment aged <5 years, and *F*/*T* ratios decreased in children with evidence of fibular overgrowth. For children who initiated treatment aged ≥5 years, tibial bowing did not worsen compared with baseline, and the results showed trends toward improvement in overall and distal fibula overgrowth. Altogether, these 1-year placebo-controlled results suggest that vosoritide treatment may improve or limit the progression of genu varum in children with achondroplasia.

## Data Availability

Restrictions apply to the availability of some or all data generated or analyzed during this study to preserve patient confidentiality or because they were used under license. The corresponding author will on request detail the restrictions and any conditions under which access to some data may be provided. The deidentified individual participant data that underlie the results reported in this article (including text, tables, and figures) will be made available together with the research protocol and data dictionaries, for noncommercial, academic purposes. Additional supporting documents may be available upon request. Investigators will be able to request access to these data and supporting documents via a data sharing portal beginning 6 months and ending 2 years after publication. Data associated with any ongoing development program will be made available within 6 months after approval of relevant product. Requests must include a research proposal clarifying how the data will be used, including proposed analysis methodology. Research proposals will be evaluated relative to publicly available criteria available at https://www.biomarin.com/publication-data-request/ to determine if access will be given, contingent upon execution of a data access agreement with BioMarin Pharmaceutical Inc.

## Clinical trial information

Clinical trial registration: NCT03583697 and NCT03197766
